# Caffeinated Skincare Development and Optimization: Upcycling Spent Coffee Grounds Into Natural Exfoliants

**DOI:** 10.1111/jocd.70607

**Published:** 2025-12-13

**Authors:** Alisa Elezović, Belma Pehlivanović Kelle, Amila Oras, Hava Mujkić, Meldina Memić, Elis Kojčin, Merima Bulbulušić, Alen Mujčinović, Aleksandra Nikolić, Fahir Bečić

**Affiliations:** ^1^ Department of Pharmaceutical Technology, Faculty of Pharmacy University of Sarajevo Sarajevo Bosnia and Herzegovina; ^2^ Department of Pharmacology and Clinical Pharmacy, Faculty of Pharmacy University of Sarajevo Sarajevo Bosnia and Herzegovina; ^3^ Department of Food Technologies, Faculty of Agriculture and Food Science University of Sarajevo Sarajevo Bosnia and Herzegovina; ^4^ Department of Agri‐Food Economics, Faculty of Agriculture and Food Science University of Sarajevo Sarajevo Bosnia and Herzegovina

**Keywords:** environmental impact, exfoliant formulation, skin sensory assessment, spent coffee grounds, transepidermal water loss

## Abstract

**Background:**

Spent coffee grounds (SCG) offer significant ecological and cosmetic potential. The conversion of SCG into safe and efficient cosmetic products promotes all aspects of sustainability and circular practices within the cosmetic industry.

**Aims:**

This study aimed to show the interdisciplinary bottom‐up approach within two focal points: demonstrating the use of upcycled materials in high‐value natural products and understanding how base formulations' content and properties influence the characteristics, consumer preferences, and clinical efficacy of exfoliant products.

**Methods:**

Methods included characterization of SCG, development of formulations, physicochemical characterization, stability testing, and the estimation of carbon dioxide emissions of the preparation process. Consumer panel testing optimized the most preferred sensory characteristics of exfoliant products chosen for clinical evaluation. Transepidermal water loss measurements assessed skin barrier disruption and recovery after the use of exfoliant formulations and after the application of hand cream.

**Results:**

The SCG found richest in bioactive substances was incorporated in three concentrations into seven exfoliant formulation bases. Stable formulations were assessed by a consumer panel, and four formulations with the best sensory characteristics were ointment‐based (F1) with 5% and 10% SCG, cream‐based (F6), and gel‐based (F11) with 5% SCG. The ointment‐based exfoliant showed no significant changes after application or after hydration cream. Aqueous formulations (F6 and F11) temporarily disrupted skin barrier function, but full recovery occurred within 6 h of hand cream application.

**Conclusion:**

By utilizing SCG, high‐quality cosmetic formulations can be created that are well‐received by consumers. Clinical findings suggest that different exfoliant formulations may suit various skin types.

## 1 Introduction

The cosmetics industry has steadily increased over time, attaining a significant global value. Skin care products represent the most significant segment of the cosmetics industry, with a market share of nearly 40% and projected to generate revenues surpassing US$236 billion by 2030 [1]. Additionally, there is a significant trend towards sustainability among consumers, with a growing preference for natural products and those promoted as “chemical‐free,” organic, and plant‐based [2]. The necessity to meet Sustainable Development Goals (SDGs) is becoming more pressing, leading the cosmetic industry to explore innovative and sustainable solutions that aim to lessen its negative effects on the environment, economy, and society [3]. Therefore, it is important to address the necessity to meet SDG goals by analyzing and improving industries, distributors, and consumers' behavior and readiness to provide a fair and equitable system. This can be achieved through various models of circular economy that could support the cosmetic industry in the transition towards a sustainable future [4].

Concerning environmental and sustainability issues of the cosmetic industry, agricultural and food processing, the circular bioeconomy of coffee is worth being challenged from sustainable manufacturing and environmental aspects [5]. The coffee fruit is of notable ecological and cosmetic relevance, with its various elements sourced from bean processing currently being employed. Additionally, the remnants produced after the preparation and enjoyment of coffee are of interest, available in both powdered form and as an extract [6]. Although they are already used in commercial products, there are few published studies on the development and characterization of coffee grounds products [7, 8]. According to Johnson et al. [9], only 30% of the mass of coffee beans can be extracted for beverage production, leaving a significant portion as insoluble waste [9]. Consequently, the coffee processing industry, which comprises over 2000 distinct components, generates substantial amounts of valuable waste [6]. It is estimated that 500 billion cups of coffee are consumed daily worldwide, and for every ton of brewed coffee, approximately 650 kg of grounds are produced [10, 11]. Population size has a significant influence on consumption levels [12]. However, despite its small population size, Bosnia and Herzegovina ranked among the top 10 in per capita coffee consumption, with 46 000 t consumed and a per capita consumption rate of 14.4 kg/capita in 2022 [13]. As research into circular business models expands, various uses for this waste are being identified, including the production of biofuels [14], bio‐oil, and ingredients for natural cosmetics [15]. The primary components of spent coffee grounds (SCG) that provide significant value and opportunities for further use are bioactive compounds, which include minerals, polyphenols, and antioxidants. These bioactive substances have beneficial properties such as antioxidant, anticancer, antiallergic, anti‐inflammatory, and antitumor effects [16]. Tannins, a major group of polyphenolic compounds obtained from coffee byproducts, are particularly valued in the beauty industry for their antioxidant properties and ability to reduce the appearance of enlarged pores on the skin. This function is crucial in enhancing the health and visual attractiveness of the skin. Considering the value of the mentioned components, a significant boom in the technological development of value‐added products will increasingly be directed towards products enriched with antioxidants and polyphenols from SCG, which is partly encouraged due to the EU Directive 2018/851 [9]. The transformation of SCG into safe and effective cosmetic products not only creates added value for all stakeholders but also fosters all dimensions of sustainability and circular practices within the cosmetic sector [17].

Recent studies have shown that SCGs can be effectively incorporated into a variety of cosmetic formulations such as facial and body exfoliants, emulsions, hydrogels, and sunscreens, providing both functional performance and sensory appeal [6, 8]. In particular, the use of SCG particles as natural exfoliants offers an environmentally friendly alternative to synthetic microbeads, while SCG‐derived oils have exhibited strong efficacy in improving skin texture and reducing signs of aging [18]. The integration of SCGs into cosmetic products thus not only valorizes an abundant food industry by‐product but also supports the development of high‐performance, eco‐conscious formulations [8]. It is suggested by Desi et al. [19] that SCG can be used as an abrasive agent in cosmetic scrubs, resulting in products that not only emit a pleasant coffee fragrance but also boast a natural and eco‐friendly origin, a feature that consumers highly value. Scrubs and exfoliants are cosmetic formulations designed to eliminate dead skin cells from their surface, which are fundamental to skin care and cleansing. Exfoliation refers to the process of eliminating the dead outer layer of the epidermis from the skin's surface. This procedure can be achieved through methods such as microdermabrasion, as well as chemical and mechanical peelings [20]. Mechanical peels are particularly popular due to their simplicity and the possibility of performing them at home. Formulations of such peelings have a semi‐solid consistency of cream or gel and necessarily contain abrasive granules [21]. Until recently, these materials were predominantly synthetic, which is suitable considering their inert properties as well as the dimensions and morphology of the particles. However, these substances present a considerable environmental challenge, as they serve as a prime example of microplastics, which are acknowledged as a significant pollutant [16, 22]. In the United States, their utilization is banned, while the European Union is proactively working on establishing regulations to restrict the deliberate inclusion of microplastics in cosmetics and other products. Possible alternatives are particles of suitable characteristics from natural mineral and plant sources. Employing natural abrasive particles like SCG is an effective strategy for mitigating environmental challenges and pollution [10, 23].

The main objective of this study was to achieve a technological transformation and optimization of coffee ground waste, converting it into a highly valuable bioactive raw material that can be utilized as a foundational component in the formulation of cosmetic products. Also, the study aimed to conduct a clinical evaluation of the sensory characteristics and efficacy of the selected formulations on healthy individuals, thereby validating the quality of the product and the legitimacy of circular practices.

Additionally, it is significant to emphasize that the aims of this study directly align with SDG 12 (Responsible Consumption and Production) by promoting sustainable resource utilization and waste minimization, as well as SDG 3 (Good Health and Well‐being) through the incorporation of potentially safer, natural ingredients while offering opportunities for green entrepreneurship and job creation in support of SDG 8 (Decent Work and Economic Growth). The study also contributes to SDG 13 (Climate Action) by decreasing greenhouse gas emissions related to organic waste disposal. Through innovation in products and the establishment of sustainable alternatives in the cosmetics industry, the study also addresses SDG 9 (Industry, Innovation and Infrastructure). Furthermore, SCG can be converted into valuable products such as biofuels, bioplastics, natural fertilizers, and cosmetic ingredients, supporting SDG 7 (Affordable and Clean Energy) through renewable energy sources. Using SCG in sustainable materials helps protect ecosystems, supporting SDG 14 (Life Below Water) and SDG 15 (Life On Land) by reducing environmental harm and conserving natural habitats.

## 2 Materials and Methods

### 2.1 Materials

Spent coffee grounds were gathered from local coffee shops (Sarajevo, Bosnia, and Herzegovina) following the preparation of coffee and espresso on coffee maker machines. The coffee grounds were obtained from the following manufacturers: Selezione Red & Green—Roasted coffee beans (C1, Julius Meinl Austria GmbH, Austria), Manuel Espresso—Aroma Classico (C2, Manuel Caffe, Italy), and Espresso premium—blend of roasted coffee (C3, Vispak d.d., Bosnia and Herzegovina). The following materials were used for the development of formulations: shea butter (Comcen, Fratelli Parodi, Italy), cocoa butter (Fagron, Croatia), coconut oil (GA‐ME‐HA d.o.o., Bosnia and Herzegovina), macadamia oil (Fagron, Croatia), sunflower oil (GA‐ME‐HA d.o.o., Bosnia and Herzegovina), jojoba oil golden virgine (Textron, Spain), Triheptanoin (and) C13‐15 alcane (LEXFEEL WOW‐A, Inolex, USA), Isopropyl miristate (Sigma‐Aldrich Chemie GmbH, Germany), Cetyl ricinoleate (TEGOSOFT CR, Evonik Industries AG, Germany), Ethylhexyl stearate, octyl stearate (TEGOSOFT OS, Evonik Industries AG, Germany), Cetearyl olivate (and) sorbitan olivate (OLIVEM 1000, Hallstar, Italy), PEG 30 glicerol stearate (TAGAT S, Evonik Industries AG, Germany), Gliceryl stearate citrate (AXOL C 62, Evonik Industries AG, Germany), Cetearyl alcohol (Evonik Industries AG, Germany), Gliceryl monostearate (TEGIN M, Evonik Industries AG, Germany), Polygliceryl‐2 sesquioleate (DERMOFEEL GO SOFT, Evonik Industries AG, Germany), acacia gum and xanthan gum (SOLAGUM AX, Seppic, France), Glicerol (Centrohem d.o.o., Serbia), Phenoxyethanol and sorbitane caprylate (NIPAGUARD SCP, Clariant Produkte GmbH, Germany). Deionized water was obtained on Arium 611, Sartorius (Sartorius Lab Instruments GmbH & Co. KG, Göttingen, Germany).

### 2.2 Characterization of Spent Coffee Grounds

As soon as collected, the SCG samples were dried in a drying oven (Memmert BE500, Germany) at 60°C until constant weight (< 8%, w/w) [24]. Particle size distribution measurements were conducted with a laser‐diffraction technique on a Mastersizer 3000 with an Aero S dispersion unit and analyzed using the Mastersizer 3000 software version 3.81 (Malvern Panalytical, UK). Obscuration level was 1.41–1.90, and the compressor air pressure was 350 kPa. The results are presented as volumetric particle size distributions with diameters *d*(0.1), *d*(0.5), and *d*(0.9). The values of the volumetric diameters represent the percentage of particles having a diameter equal to or smaller than a given value of 10%, 50%, and 90%, respectively. The pH level of the samples was measured using a pH meter (Mettler Toledo, Switzerland) [25]. Functional properties of SCG—water and oil holding capacity, as well as emulsifying activity and emulsion stability, were examined according to methods described by Ballesteros et al. [26]. Quantitative estimation of tannin content was performed by the titrimetric method with standard potassium permanganate solution [27]. The total phenols content (mg GAE/g dry matter) in SCG was measured using the colorimetric method with Folin–Ciocalteu reagent (Biochrom Libra S50 UV/Vis spectrophotometer, UK), while the antioxidant activity (mmol Fe^2+^/g dry matter) was determined by the ferric reducing antioxidant power (FRAP) assay [28].

### 2.3 Preparation of Formulations

Base formulations were prepared by separately heating phases A and B (composition of individual formulations in Table 1) to 70°C and mixed at 1200 rpm (GakoUnguator, Fagron, Germany). During the cooling stage (40°C), phase C was added, and the mixture was cooled down to room temperature. An initial characterization of these formulations was conducted, leading to the exclusion of those that exhibited instability in subsequent evaluations from further exfoliant development. The formulations that demonstrated stability were then utilized to prepare exfoliant formulations by incorporating SCG powder (Vispak, B&H) at concentrations of 2%, 5%, or 10%.

**TABLE 1:** Base formulations' composition.

	Ointments			Emulsions							Gels	
	1	2	3	4	5	6	7	8	9	10	11	12
Phase A												
*Butyrospermum Parkii* Butter	+	+	+	+	+		+		+	+		
*Cocos nucifera* (cocount) oil	+		+	+		+	+	+		+		
*Theobroma cacao* seed butter		+			+	+		+	+			
*Macadamia ternifolia* seed oil	+			+				+		+		
*Helianthus annuus* seed oil					+		+	+				+
*Simmondsia chinensis* (jojoba) seed oil		+			+		+		+	+	+	
Triheptanoin (and) C13‐16 isoalkane			+			+	+		+			
Isopropyl myristate	+			+								
Cetyl ricinoleate		+			+			+		+		
Ethylhexyl stearate, octyl stearate			+			+			+			
Cetearyl olivate (and) sorbitan olivate	+	+	+	+			+			+	+	
PEG 30 glycerylstearate					+							+
Gliceryl stearate citrate						+		+	+			
Cetearyl alcohol				+	+		+					
Glyceryl stearate				+	+					+		
Polyglyceryl‐2 sesquioleate						+			+			
Phase B												
Glycerol				+	+	+	+	+	+	+	+	+
*Acacia Senegal* gum (and) xanthan gum				+	+	+	+	+	+	+	+	+
Aqua				+	+	+	+	+	+	+	+	+
Phase C												
Phenoxyethanol (and) sorbitan caprylate				+	+	+	+	+	+	+	+	+

### 2.4 Physicochemical Characterization of Formulations

The physicochemical properties of both base formulations and exfoliant formulations were determined at least 24 h after the preparation of the formulations. They included organoleptic properties (physical appearance, color, homogeneity, and consistency), determination of pH value, spreadability, and mechanical stress. The pH value was determined using a pH meter (SevenEasy, Metler Toledo GmbH, Germany) at room temperature (25°C). In order to determine spreadability, 0.5 g of each formulation was weighed on the analytical balance, AG 400 (Metler Toledo GmbH, USA), and placed on the glass plate inside the marked circle with a diameter of 1 cm. A second glass plate was placed over the first, and a 200 g weight was placed on it. After 2 min, the diameter (expressed in centimeters) was measured across 4 diagonals, and the average was recorded. The analysis was performed at room temperature (25°C) in triplicate [29].

The physical stability (mechanical stress) of formulations was determined by centrifugation. A total amount of 1.3 g of each formulation was weighed. Then, samples were centrifuged (Hettich Mikro 22R, Andreas Hettich GmbH & Co. KG, Germany) at 3000 rpm for 30 min at 25°C [30]. The analysis was performed in triplicate. Due to the interference of SCG particles, only base formulations were characterized by microscopy. The base emulsion formulations were examined using the inverted microscope (VisiScope IT415 PH with VisiCam P6, VWR International LLC, Italy) to assess their microstructure, droplet consistency, and stability.

#### 2.4.1 Stability Test

The heat shock stability of base formulations and selected exfoliant formulations with 2%, 5%, and 10% SCG were evaluated via the heating and cooling cycles, that is, four alternating cycles of cooling at 4°C (in refrigerator, Beko, Arçelik A.Ş, Turkey) for 24 h and heating at 45°C (in oven, Memmert BE500, Germany) for 24 h. The assessment was conducted to analyze the physicochemical properties of the formulations, including potential phase separation, sedimentation of spent coffee grounds (SCG), and alterations in organoleptic characteristics or consistency [31]. After subjecting all formulations to thermal cycles, assessments of organoleptic properties, pH value, spreadability, and mechanical stress were conducted as described above.

### 2.5 Assessment of the Environmental Impact of the Use of Spent Coffee Grounds (SCG) in Cosmetics Production: Analysis of CO_2_ Emissions During the Production of Exfoliant Formulations

The cosmetics industry is facing increasing pressure to reduce its environmental footprint, and this includes assessing carbon dioxide (CO_2_) emissions at all stages of production, from raw materials to the final product. Estimating the CO_2_ footprint of the exfoliant formulation will help us understand how much carbon dioxide is generated during the preparation and formulation of the exfoliant and which are the main stages of the process that generate CO_2_ emissions. To calculate CO_2_ equivalent emissions, a formula is used to convert greenhouse gas (GHG) emissions as the product of activity data (e.g., electricity consumption) and the corresponding emission conversion factor. Carbon dioxide emissions are the most important in this context because CO_2_ is emitted during the production and consumption of electricity, especially when the energy comes from fossil fuels [31]. This approach is based on official data and conversion factors from the DEFRA GHG Conversion Factors 2023, which are used to calculate emissions of all relevant GHG gases (Equation 1), as well as to calculate emissions separately for each gas (CO_2_, CH_4_, N_2_O, and others).

(1) $$E_{{\mathrm{CO}}_{2}}\left(\mathrm{kg}\right)=E\left(\mathrm{kWh}\right)\times F_{{\mathrm{CO}}_{2}}\left({\text{kgCO}}_{2}/\mathrm{kWh}\right)$$

where $E_{{\mathrm{CO}}_{2}}$ is CO_2_ emission, *E* is energy, and *F* is emission factor.

Based on the DEFRA GHG Conversion Factors 2023, CO_2_ emissions from the consumption of electric energy in the UK for the year 2023 is *F* = 0.20493 kg CO_2_/kWh.

Formula for calculating electricity consumption (Equation 2):

(2) $$E\;\left(\mathrm{kWh}\right)=P\;\left(\mathrm{kW}\right)\times t\;\left(h\right)$$

where *E* is energy, *P* is power, and *t*, time. This formula is used to calculate the consumed electrical energy when the power of the device and the operating time are known. It is used as input data in models for estimating greenhouse gas emissions.

**FIGURE 1:**
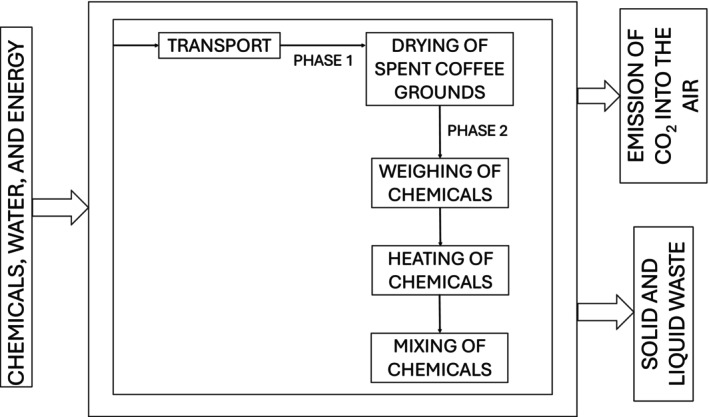
LCA system boundaries: Stages of the exfoliant formulations manufacturing process suitable for CO_2_ emission analysis (T‐transport).

The system boundaries used in Life Cycle Assessment (LCA) are presented in Figure 1.

### 2.6 Clinical Evaluation and Participants

The clinical evaluation was performed as a double‐blind, randomized, controlled trial to assess sensory characteristics and effects on skin barrier function of selected SCG formulations. The trial was conducted in February 2025 at the University of Sarajevo—Faculty of Pharmacy, following the principles of the Declaration of Helsinki. The Ethical Committee of the University of Sarajevo—Faculty of Pharmacy approved the research protocols (approval numbers: 0101‐6838/24 and 0101/6837/24) in December 2024. A public workshop entitled “Let's unlock the potential of secondary resources through circular business models—examples of coffee grounds and high‐value cosmetic products” outlined study protocols, methodologies, and overall aims and served as a platform for participant recruitment. In total, 32 participants (both male and female) were included in the study and divided into two separate study sessions: (I) sensory panel evaluation and (II) measurement of transdermal water loss. Written informed consent was obtained from all participants who met the following inclusion criteria: (1) willingness to participate based on their own free will, (2) sign an informed consent, (3) age of 18 years or older at the time of conducting the study, (4) good health status, (5) absence of known allergies and skin diseases, (6) no aversion to exfoliants, coffee, or products with upcycled ingredients. Exclusion criteria were the following: (1) any potential conflict of interest that could affect participation, (2) allergies, (3) skin diseases, damage, and/or skin hypersensitivity, (4) aversion to exfoliants, coffee, or products with upcycled ingredients.

#### 2.6.1 Sensory Panel Evaluation

This panel evaluation sought to compare the most effective formulations of exfoliants containing SCG and to identify those formulations that exhibit optimal sensory characteristics as perceived by untrained consumers for further development. A total of 21 untrained participants who met the specified inclusion criteria participated in this study session. Each participant was requested to complete a self‐assessment questionnaire, which was divided into two sections: (1) demographic characteristics with consumer habits and perspectives and (2) sensory panel evaluation. Each item (formulation) of the sensory panel questionnaire was scored on a 5‐point scale, and participants numerically graded all 9 formulations based on their scent (intensity and preference), appearance, and texture (texture, pick‐up, spreadability, abrasiveness, skin adherence, skin feel after application, ease of removal, skin after‐feel). Participants evaluated 9 different formulations (comprising 8 SCG formulations and a similar commercial product) according to the previously established and approved instructions. The tested formulations contained coffee grounds in different formulations (5% and 10%) and were distributed differently in random order on individual transparent plastic trays under distinct codes. The tested formulations were compared with a similar product available from the Bosnian market (Nacomi Body Exfoliant Iced Coffee, Nacomi, Poland).

#### 2.6.2 Measurement of Transepidermal Water Loss

The effects of SCG formulations on skin barrier function were evaluated by determining transepidermal water loss (TEWL), also known as *perspiration insensibilis*. A total of 20 participants who met the specified inclusion criteria participated in this study session. Participants were instructed not to apply any products on the skin at least 24 h before the study, as well as to avoid any physical activity before the measurement. Before the measurements, all participants underwent acclimatization for 10 min.

The four formulations that received the highest scores in prior sensory panel evaluations were selected for the TEWL measurement. This evaluation was performed in a separate session.

All measurements were conducted by the same examiner, who had previously received training on the proper use of the meter. The VapoMeter closed‐chamber device (Delfin Technologies Ltd., Finland) was used for measurement of TEWL at five previously established sites (4 SCG formulations and control) on the left and right volar forearms. At the beginning of the study, the TEWL was measured on all 5 sites (1st measurement time point—initial baseline measurement). The exfoliant formulations were applied on four sites, and one site was left as control. Each formulation was applied by rubbing it for 10 s and then rinsed off with a wet towel by the same operator, and the TEWL was measured for the second time (2nd time point). The same moisturizing cream (Norwegian Formula Fast Absorbing Hand Cream, Nongreasy, Neutrogena, France) was applied on all sites. The TEWL values were measured after 2 h (3rd time point), 4 h (4th time point), and 6 h (5th time point) post‐application of the cream. All measurements were taken at room temperature (20°C ± 2.5°C) and relative humidity of 34% ± 3.5%. The measurements were normalized: firstly, in comparison to the control site, by dividing each value by the control site value at the same time point and secondly, in comparison to baseline measurement (M1) by dividing each already normalized value by the value of the same site at baseline. The obtained TEWL values were indicative of the barrier function disruption after application and rinse‐off of the exfoliant formulations, but also barrier function restoration over 6 h after the application of the hydration cream.

Additionally, all participants completed a self‐assessment questionnaire divided into four sections: (1) demographic information, (2) skin characteristics—evaluation before application, (3) skin characteristics—evaluation immediately after application, and (4) skin characteristics—evaluation 6 h post‐application.

### 2.7 Statistical Analysis

Statistical analysis included descriptive analysis (means and standard deviations—SD calculations) and univariate statistics. The impact of coffee brand (independent variable, three levels) on the examined physico‐chemical parameters (dependent variable) of the SCG samples was evaluated using one‐way analysis of variance (ANOVA). One‐way ANOVA was also used to examine the effect of SCG share (independent variable, four levels) in the formulation on its spreadability (dependent variable), as well as the effect of SCG share (independent variable with three levels) on spreadability after the applied thermal stress (dependent variable). The same analysis was used to examine the impact of the assessment period (independent variable, three levels) on the sensory characteristics (dependent variable) of the participants' skin. Statistically significant differences among samples were further examined by Tukey's *post hoc* test at *p* ≥ 0.05. Additionally, a *t*‐test was performed to examine the presence of significant differences in spreadability before and after thermal stress among samples with the same SCG content and within each formulation. Descriptive analysis was performed using MS Office Excel, while univariate statistical analyses were carried out using PAST software (4.17c, 2024).

## 3 Results and Discussion

### 3.1 Characteristics of Spent Coffee Grounds

All three SCG samples showed similar unimodal particle distribution as presented in Table 2, indicating a consistent grind type, typical for espresso or moka pot grind. The C1 sample exhibited the finest and most uniform particle size distribution, with a median particle size (Dx50) of 304.5 μm. In contrast, the C2 sample had the coarsest grind (Dx50 = 362 μm), while C3 showed intermediate values (Dx50 = 313 μm). Mean particle size diameters were similar to those reported by Brekalo et al. [32], ranging from 200 to 400 μm. Smaller and more uniform particles, as observed in C1 and C3 samples, are positively correlated with enhanced functional properties, including water holding capacity (WHC), oil holding capacity (OHC), emulsifying activity (EA), and emulsion stability (ES). These functional properties are attributed to increased surface area and interfacial interaction, allowing for more efficient physical entrapment and emulsification. The obtained results are in accordance with those reported by Ballesteros et al. [26].

**TABLE 2:** Physical and chemical parameters of analyzed SCG samples (±SD).

	C1	C2	C3
Physical parameters			
Particles distribution μm			
*d*(0.1)	63.5 ± 5.2	84.8 ± 22.9	51.4 ± 7.99
*d*(0.5)	304.5 ± 13.4	362 ± 21.2	313 ± 4.2
*d*(0.9)	594 ± 62.2	699.5 ± 65.8	585.5 ± 0.71
Particle specific surface area (m^2^/kg)	37.4 ± 2.6	28.3 ± 5.98	46.1 ± 3.4
Water holding capacity (g water/g dry sample)	5.95 ± 0.03^a^	5.31 ± 0.02^b^	5.47 ± 0.03^c^
Oil holding capacity (g oil/g dry sample)	5.85 ± 0.02^a^	5.2 ± 0.03^b^	5.69 ± 0.03^c^
Emulsifying activity (%)	57.0 ± 0.04^a^	50.4 ± 0.02^b^	55.2 ± 0.03^c^
Emulsion stability (%)	90.1 ± 0.04^a^	84.3 ± 0.05^b^	89.7 ± 0.06^c^
Chemical parameters			
pH value	5.4 ± 0.05^a^	5.5 ± 0.03^b^	5.3 ± 0.02^c^
Tannins content (%)	1.38 ± 0.2^a^	1.6 ± 0.07^b^	1.65 ± 0.08^c^
Total phenols content (mg GAE/g dry sample)	10.1 ± 0.05^a^	14.2 ± 0.2^b^	17.7 ± 0.1^c^
Antioxidant activity (mmol Fe^2+^/g dry sample)	0.049 ± 0.1^a^	0.076 ± 0.1^b^	0.17 ± 0.09^c^

*Note:* Different letters (a–c) in rows for each analyzed parameter represent a statistically significant difference between the means of SCG samples (Tukey's test, *ρ* ≥ 0.05).

The chemical characterization of the SCG samples revealed notable differences in their pH, tannin content, total phenolic content, and antioxidant activity. All samples exhibited slightly acidic pH values ranging from 5.3 to 5.5, indicating suitability for applications where mild acidity is desirable, such as in food, pharmaceutical, or cosmetic formulations. The C3 demonstrated the highest levels of bioactive compounds, with a tannin content of 1.65%, total phenolic content of 17.7 mg GAE/g dry matter, and antioxidant activity of 0.170 mmol Fe^2+^/g dry matter. Similar contents of total phenols were reported by other researchers [33, 34] as well as antioxidant activity [28]. These findings suggest that C3 possesses a superior capacity for free radical scavenging and oxidative stress mitigation, making it a strong candidate for applications targeting antioxidant functionality. However, the C1 sample's slightly lower pH and simpler phenolic profile may be advantageous in formulations where lower reactivity or less bioactivity is required.

### 3.2 Physicochemical Characteristics of the Formulations

**FIGURE 2:**
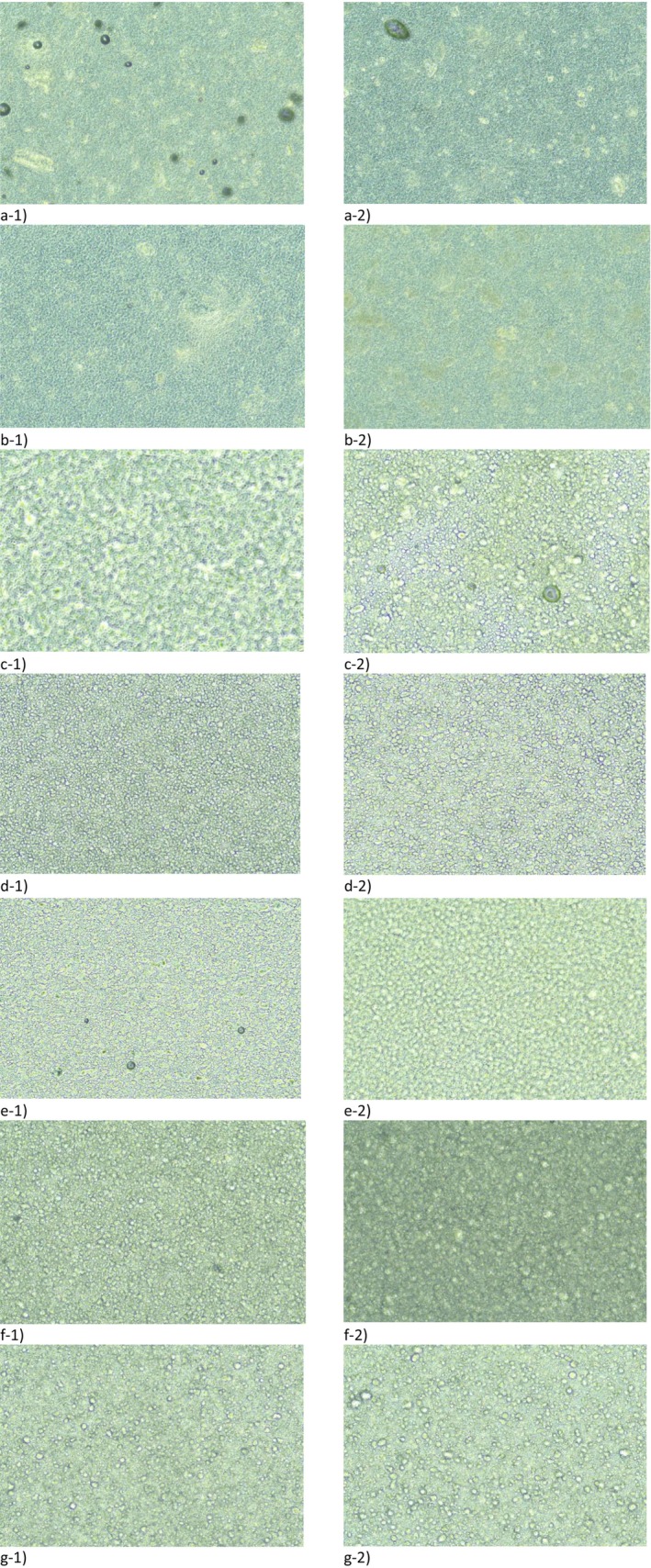
Microscopy images of selected base formulations before (1) and after (2) thermal stress testing at 20× magnification: (a) F1, (b) F3, (c) F4, (d) F6, (e) F9, (f) F10, (g) F11.

In the first stage, the base formulations without SCG were assessed for their stability and compatibility of ingredients. The formulations didn't change their appearance over time, but some of them were unstable either upon mechanical stress (centrifugation) or after thermal stress testing or upon centrifugation following thermal stress testing. Thus, formulations F1, F3 (anhydrous ointment‐based); F4, F6, F9, F10 (cream‐based); and F11 (gel‐based) were chosen as stable for further studies. Their photographs under an inverted microscope at 20× magnification are shown in Figure 2 before and after thermal stress testing, showing minimal changes. In these selected formulations, the SCG chosen as described above was incorporated at either 2%, 5%, or 10% and further tested.

All chosen aqueous formulations had satisfactory pH values [35] that were stable pH values and did not vary upon the addition of SCG. Also, the percentage of SCG did not affect the pH values, which were stable upon thermal stress cycles, varying at most by 0.3, with very small variation between the samples (Table 3). In this way, it can be concluded that basic compatibility and chemical stability were achieved. The spreadability values of the base formulations were generally higher than those after the addition of SCG. However, once SCG was added, the values of all formulations were roughly 4 and had a gradual decrease with the increase in SCG concentration, but by at most 0.59 cm (F10) (Table 4). After thermal stress cycles, the spreadability remained constant (differences were < 5%, statistically significant only in formulation F3‐5%, F4‐5%, and F10‐10%), indicating the stability of the internal structure of exfoliant formulations.

**TABLE 3:** Average (±SD) pH values of exfoliant formulations before (*n* = 1) and after (*n* = 3) thermal stress test.

Formulation	pH value				pH value after thermal stress		
	Percent SCG						
	0%	2%	5%	10%	2%	5%	10%
F4	5.56	5.56	5.54	5.49	5.31 ± 0.01	5.19 ± 0.01	5.18 ± 0.03
F6	5.00	4.97	5.01	5.07	4.73 ± 0.02	4.80 ± 0.01	4.85 ± 0.01
F9	5.00	5.02	5.05	5.09	4.84 ± 0.01	4.85 ± 0.01	4.89 ± 0.02
F10	5.56	5.57	5.58	5.47	5.39 ± 0.08	5.29 ± 0.03	5.20 ± 0.01
F11	6.02	5.78	5.68	5.61	5.55 ± 0.02	5.47 ± 0.03	5.32 ± 0.01

**TABLE 4:** Average (±SD) spreadability of exfoliant formulations before and after thermal stress test (*n* = 3).

Formulation	Spreadability (cm)				Spreadability after thermal stress (cm)		
	Percent SCG						
	0%	2%	5%	10%	2%	5%	10%
F1	4.93 ± 0.25^a^	4.33 ± 0.20^b^	4.23 ± 0.06^b^	4.01 ± 0.09^b^	4.36 ± 0.14	4.28 ± 0.14	4.11 ± 0.15
F3	6.57 ± 0.06^a^	4.33 ± 0.13^b^	4.13 ± 0.04^c^	3.96 ± 0.05^c^	4.22 ± 0.07^x^	3.97 ± 0.05^xy^*	3.83 ± 0.18^y^
F4	7.20 ± 0.05^a^	4.37 ± 0.06^b^	4.22 ± 0.03^c^	3.95 ± 0.03^d^	4.42 ± 0.05^x^	4.03 ± 0.02^y^*	3.80 ± 0.05^z^
F6	7.75 ± 0.13^a^	4.40 ± 0.05^b^	4.05 ± 0.03^c^	3.82 ± 0.04^d^	4.41 ± 0.08^x^	4.09 ± 0.04^y^	3.73 ± 0.07^z^
F9	8.01 ± 0.21^a^	4.41 ± 0.06^b^	4.05 ± 0.08^c^	3.87 ± 0.05^c^	4.36 ± 0.05^x^	3.97 ± 0.03^y^	3.83 ± 0.03^z^
F10	6.89 ± 0.04^a^	4.41 ± 0.06^b^	4.06 ± 0.06^c^	3.82 ± 0.04^d^	4.26 ± 0.14^x^	3.96 ± 0.01^y^	3.66 ± 0.09^z^*
F11	7.24 ± 0.08^a^	4.37 ± 0.04^b^	4.02 ± 0.11^c^	3.85 ± 0.03^c^	4.37 ± 0.03^x^	4.06 ± 0.05^y^	3.78 ± 0.04^z^

*Note:* Different letters in rows for each formulation indicate statistical significance in spreadability (letters a–d) and spreadability after thermal stress (letters x–z) among samples with different shares of SCG (Tukey's test, *ρ* ≥ 0.05). An asterisk (*) indicates statistically significant difference in spreadability before and after thermal stress between samples with the same SCG content per each formulation (*t*‐test, *ρ* ≤ 0.05).

The mechanical stress test was performed after the thermal stress test. As can be seen from Table 5, formulations F1, F4, and F10 remained stable, without phase separation or even sedimentation of the SCG particles. Sedimentation of SCG particles was not considered a sign of instability, but rather a sign of the lighter base formulations, which might allow some sedimentation on prolonged storage. Phase separation was observed most notably in formulations F3 and F9, disqualifying these formulations from further development stages. F6 showed only sedimentation at 5% SCG, but at a high concentration of SCG of 10%, there was also some phase separation. When comparing this formulation with F4 and F10 (very similar in excipient content), the major difference is the emulsifier system. It appears that the main hydrophobic emulsifier, a mixture of cetearyl olivate and sorbitan olivate in these two formulations, makes a significant difference in comparison to glyceryl stearate citrate in F6. The choice of lipophilic excipients (internal phase, emulsifier component, viscosity‐increasing agents) also influences the internal structure and stability of the system.

**TABLE 5:** Physical stability of exfoliant formulations after centrifugation (mechanical stress) after thermal stress test.

Formulation	Mechanical stress after thermal stress^a^			
	Percent SCG			
	0%	2%	5%	10%
F1	S	S	S	S
F3	S	O	O	O
F4	S	S	S	S
F6	S	S	T	T+O
F9	S	T+O	O	O
F10	S	S	S	S
F11	S	T	T	T

^a^ S‐Stabile, T‐Sedimentation, O‐Phase separation.

### 3.3 Ecological Impact of the Production Process

The process of SCG disposal in landfills is not as simple as most other waste materials, because due to the high organic composition of SCG, when not treated and disposed of in large quantities, there is a high risk of spontaneous combustion and release of large amounts of methane and carbon dioxide, as well as the emission of odors associated with fermentation processes. When landfill is not a suitable option, incineration of SCG is also a viable option for material disposal. However, the burning of SCG is controversial due to the emission of greenhouse gases, which contribute to pollution. Due to the growing concern about the negative impact on the environment and the pressure to reduce pollution, researchers are looking for new solutions for recycling this waste. One of them is the utilization of SCG in high‐value‐added products. The collection of the SCG is an important process to reduce the environmental footprint. In this analysis, a very small amount of SCG was used, due to which transportation costs were excluded from the calculations and considered negligible, under the principle of minimal impact in life cycle assessment (LCA). Analysis was done for the following processes (Table 6): (i) drying of SCG, and according to the analysis, 4.426 kg of CO_2_ equivalent (CO_2_e) was produced, (ii) preparing formulation, including weighing, heating, and mixing of chemicals, in total producing 0.842 kg of CO_2_e. In total, 5.268 kg of CO_2_e is produced for the transformation of used SCG into high‐value‐added products. Landfill disposal leads to significant greenhouse gas emissions, primarily due to methane production, which has a much higher global warming potential than CO_2_. Therefore, circular practices should not be neglected, especially if you consider the amount of SCG generated annually and disposed of at a landfill (i.e., some estimates 60 million tons) and contributing 504 kg CO_2_ equivalent per ton of SCG [35]. Therefore, our findings contribute to a multifaceted approach in knowledge creation, the development of scientific understanding of the environmental impact, and the possibility to utilize food waste and set up a foundation for initiatives that are fostering circular economy development. Furthermore, contribution can be seen in bridging the gap between growing awareness of the environmental impact of personal choices [36, 37, 38] and the actual application of sustainable business practices, especially for attractive and innovative sectors like HORECA, where close ties to consumers will ensure smooth and efficient communication of value by highlighting efforts to utilize waste and produce with less environmental impact. Particularly in developing and transitional economies, this may be seen as a “trigger” for more sustainable ways of producing and consuming products and improving the competitive position of the companies. Finally, insights from this study can serve as input for policymaking processes and new initiatives and partnerships to tackle current challenges and spur sustainable production and consumption.

**TABLE 6:** Analysis of CO_2_ emission.

Process	Power (W)	Time (h)	Electricity consumption (kWh)	CO_2_ emission (kg)
Phase I				
SCG drying	900	24	21.6	4.426
Phase II				
Weighting of chemicals	15	2	0.03	0.0062
Water bath heating	1000	4	4	0.819
Mixing	80	1	0.08	0.0164

### 3.4 Consumer Preferences—Sensory Panel Evaluation

All the participants signed the informed consent and completed the entire protocol. A total of 21 participants (19 females and 2 males) completed the questionnaire on demographics, consumer habits, and views. Data obtained from the demographic information form indicated the following age group distribution: 18–24 years (*n* = 9, 42.8%); 25–34 years (*n* = 5, 23.8%); 35–45 years (*n* = 2, 9.5%); and 46–55 years (*n* = 5, 23.8%). Four participants self‐assessed their skin as normal, 4 as mixed, 1 as oily, 9 as dry, and 3 as sensitive. In relation to the application of cosmetic items in daily routines, respondents indicated an average usage of 10.35 hygiene products (with a range of 8 to 16), 8.70 skincare items (ranging from 5 to 16), and 8.43 decorative products (spanning from 0 to 17). Among the participants, 8 use exfoliants/peelings every week, 4 use them monthly, and 8 use them rarely. When asked about the importance of characteristics when choosing a cosmetic product, the participants ranked (from highest to lowest overall score): active substance/functionality, afterfeel, scent, washing out, spreadability, manufacturer, texture on applying, appearance, message/subtext, and packaging of the product. When considering the preferences towards natural cosmetics or sustainable cosmetics, 12 answered affirmatively, 3 explicitly don't prefer it, 2 said it did not matter to them, while the rest did not respond.

**FIGURE 3:**
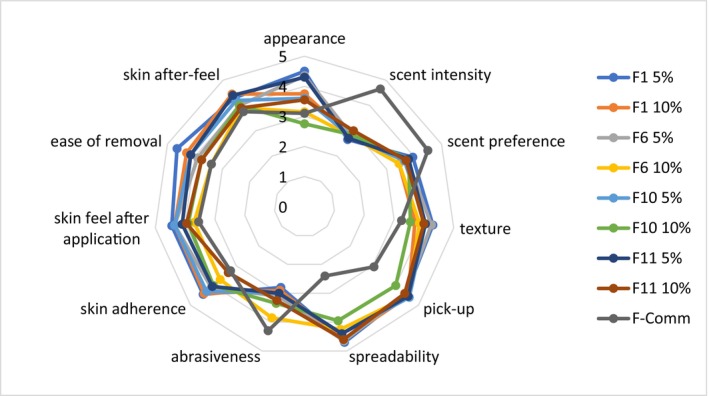
Sensory profiles of the selected exfoliant formulations (*n* = 21).

The selected formulations (F1, F6, F10, and F11) with 5% and 10% of SCG were evaluated by participants. Overall, as can be seen from Figure 3, the differences among the tested formulations were not as large as they were compared to the commercial product. The only difference between the anhydrous formulation (F1) and the commercial product was that it also contained sugar crystals as well as added scent, which is reflected in the average scores for the attributes “scent intensity”, “scent preference”, and “abrasiveness”. Also, this formulation was not very easily picked up or spreadable due to the larger particles. When comparing the developed exfoliant formulations, the appearance, texture as well as skin feel‐related attributes scores were higher for the 5% SCG containing formulations. Scent characteristics were very similar for all formulations. The formulations F1‐5%, F6‐5%, F11‐5%, and F1‐10% had the highest overall scores (sum of average values for all attributes).

**FIGURE 4:**
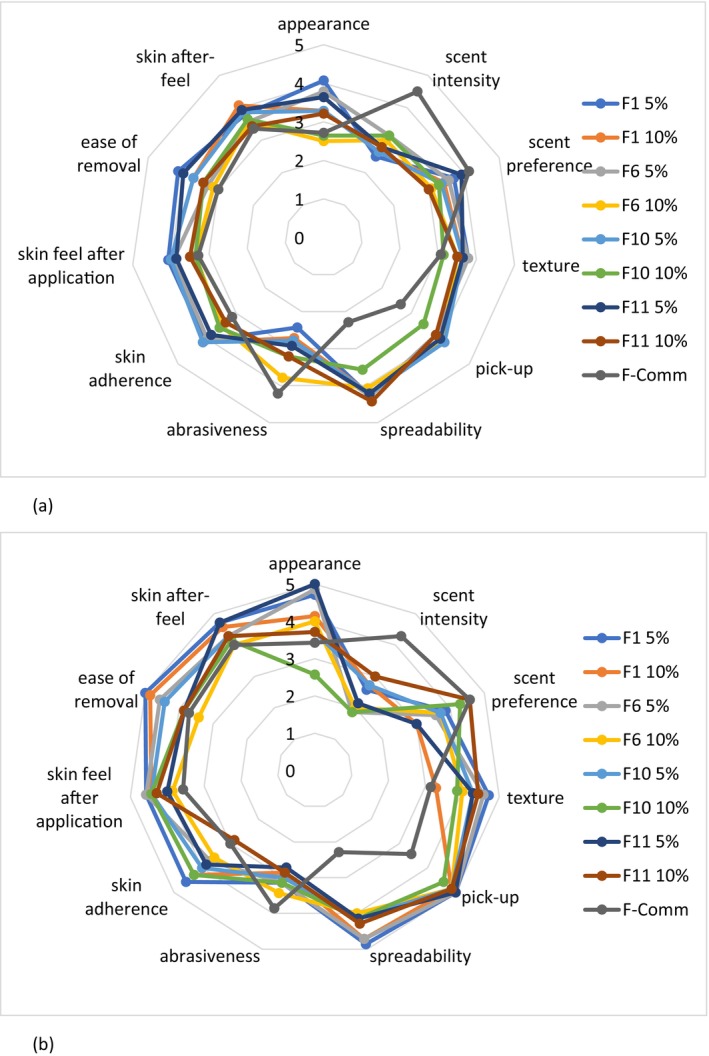
Sensory profiles of the selected formulations—age differences (a) Age groups 18–34 (*n* = 14), (b) Age groups 35–65 (*n* = 7).

When answers were grouped according to the age of participants into two groups (Figure 4), it is interesting to note that, except for scent intensity and abrasiveness attributes, the scores were higher in the older age group. The ranking for the younger age group was (higher to lower): F11‐5%, 1%–5%, 6%–5% and 1%–10%, with quite a strong preference for these four formulations (scores in the range of 40.79–39.93). In the second age group, the formulations ranked (higher to lower score): 1%–5%, 6%–5%, 1%–10%, and 10%–5%, thus preferring more emollient formulations.

The highest‐scoring formulations, F1‐5%, F6‐5%, F11‐5%, and F1‐10% were chosen for further efficacy testing.

### 3.5 Skin Barrier Disruption and Recovery Assessment

A total of 20 participants (16 females and 4 males) completed a demographic information form and a self‐assessment questionnaire concerning their skin characteristics and signed the informed consent. During the same session, measurements of TEWL were successfully performed at the specified time points for each participant.

Data obtained from the demographic information form indicated the following age group distribution: 18–24 years (*n* = 10, 50.0%); 25–34 years (*n* = 3, 15.0%); 35–45 years (*n* = 3, 15.0%); and 46–55 years (*n* = 4, 20.0%). Participants self‐assessed and categorized their skin types as follows: normal (*n* = 5, 25.0%); dry (*n* = 8, 40.0%); sensitive (*n* = 4, 20.0%); and mixed (*n* = 3, 15.0%). In total, 11 participants reported using mechanical exfoliants or chemical peelings “weekly”, while one participant indicated a “monthly” usage. Additionally, four participants mentioned using exfoliants or peelings “rarely”, and five participants stated that they do not use exfoliants or peelings whatsoever. In response to inquiries regarding the significance of various factors in selecting a cosmetic product, participants prioritized the following characteristics in descending order of overall score: manufacturer, message/subtext, product packaging, and appearance, which received the highest rating. In contrast, lower scores were assigned to scent, washability, texture during application, spreadability, afterfeel, and the effectiveness of active ingredients. When assessing attitudes towards natural and sustainable cosmetics, 10 individuals responded positively, while 5 indicated a clear disfavor, and another 5 stated that it was of no significance to them.

**FIGURE 5:**
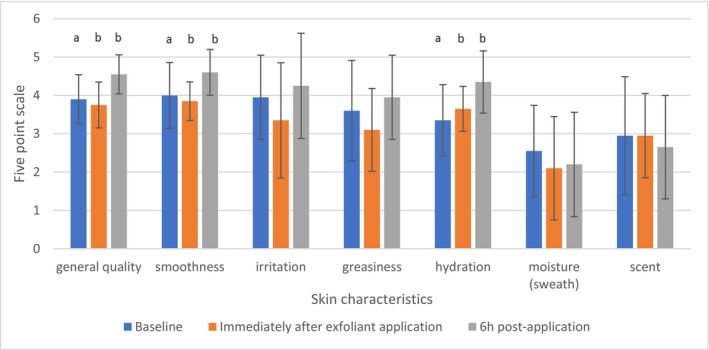
Results from the self‐assessment questionnaire on seven skin characteristics, which include assessments conducted prior to application, immediately following application, and 6 h after application. Different letters (a–b) per each evaluated skin characteristic indicate statistically significant difference between the means (Tukey's test, *ρ* ≥ 0.05).

The results from the self‐assessment questionnaire regarding skin characteristics, which included assessments conducted prior to application, immediately following application and rinse‐off, and 6 h after application, are illustrated in Figure 5. The questionnaire utilized a 5‐point scale for scoring, allowing participants to assess skin characteristics through seven distinct features: (1) overall quality, (2) smoothness, (3) irritation, (4) greasiness, (5) hydration, (6) moisture (sweat), and (7) scent. The results from the self‐assessment questionnaire on skin characteristics show an overall improvement across all measured features, with the exception of moisture (sweat) and odor. Since the study was conducted in December and the humidity was quite low, the low grades for moisture are understandable. The scent was not added to the formulations; thus, the skin was not scented post‐application. An important point to mention is that the participants were untrained, and no further instructions were given to them; therefore, this was purely their opinion on their skin characteristics. No correlations were found between the results of the questionnaire at the baseline and TEWL measurements at the baseline.

**FIGURE 6:**
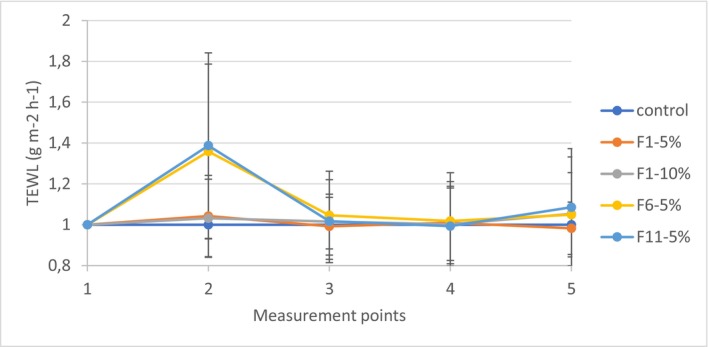
Normalized average transepidermal water loss (TEWL) values (±SD; *n* = 20) for SCG formulations and control over time points (1—baseline measurement; 2—post‐application of the formulations; post‐application of hydration cream: 3–2, 4–4, and 5–6 h).

The normalization of the raw TEWL measurement data was performed to account for slight changes in the ambient conditions and intraindividual shifts in TEWL/perspiration of the participants over the course of the study, as well as to highlight the differences in TEWL that arise from products' usage (both exfoliant formulations and moisturizing cream). As shown in Figure 6, ointment‐based exfoliant formulation (F1) did not show significant changes after the application and rinse‐off, regardless of the SCG concentration, implying that the dominant effect was created by the base, not the SCG amount. It probably did not wash away completely, regardless of the emulsifier present functioning as a mild detergent, but rather created a protective oily film over the skin. The skin barrier was not affected either by the application of the exfoliant or subsequent hydration cream for this reason. On the other hand, aqueous formulations (F6 and F11), besides SCG as a mechanical exfoliant, contain an emulsifier that also functions as a mild detergent and internal oily phase that further collects sebum and debris. Thus, skin barrier function was temporarily significantly disrupted (*p* < 0.05, compared to the control) (Figure 6). Since both formulations' levels of TEWL increase were very similar, even though the cream formulation (F6) contained a complex oily phase, this effect can be attributed to the aqueous nature of the formulations' base, not the oily phase content. After the application of the moisturizing cream, the barrier function was restored in both cases. Six hours post‐application, TEWL values showed a very small increase (statistically insignificant, calculated by comparing the baseline to both formulations), suggesting that the protective effect of the cream was over, but still the barrier function was fully recovered.

## 4 Conclusion

Considering the significant demand for sustainability and the conservation of our planet, which is acknowledged by consumers, it is crucial to investigate the potential of a circular economy within consumer industries, such as cosmetics. By utilizing spent coffee beans, which remain a valuable source of functional compounds, high‐quality cosmetic formulations can be created that are well received by consumers. This study involved the development of exfoliant formulations, which were optimized through physio‐chemical evaluations and subsequently assessed for consumer preferences and clinical effectiveness. Ultimately, the formulations demonstrated varying effects on the skin barrier, highlighting the importance of selecting the appropriate vehicle based on skin type to achieve optimal skin benefits. The findings suggest that various formulations of exfoliants may be more suitable for different skin types. For individuals with oily skin, aqueous‐based formulations such as creams or gels may prove to be more effective for cleansing. Conversely, for those with dry skin, ointment‐based exfoliant formulations could be more advantageous, as they maintain the integrity of the skin barrier, as indicated by our unchanged TEWL values.

One of the main limitations of this study is the relatively limited sample size of participants, which may affect the generalizability of the results. Furthermore, the participants did not receive specific training before the intervention, which could have impacted the consistency and precision of the findings. These elements should be considered when analyzing the outcomes of this study. Subsequent research should strive to incorporate a larger and more varied participant group to increase statistical power and wider applicability. In addition, offering standardized training or preparatory sessions for participants before the trial may assist in minimizing variability and enhancing the reliability of the data.

## Author Contributions

A.E.: conception and design, acquisition of data, analysis and interpretation of data, drafting and revising; B.P.K.: conception and design, acquisition of data, analysis and interpretation of data, drafting and revising; A.O.: acquisition of data, analysis and interpretation of data, drafting and revising; H.M.: acquisition of data, analysis and interpretation of data; M.M.: acquisition of data, analysis and interpretation of data; E.K.: acquisition of data, analysis and interpretation of data; M.B.: acquisition of data, analysis and interpretation of data; A.M.: analysis and interpretation of data; drafting and revising; A.N. and A.M.: analysis and interpretation of data, revising, final approval; F.B.: analysis and interpretation of data, revising, final approval.

## Funding

This work was supported by the Ministry of Science, Higher Education and Youth of Sarajevo Canton, Bosnia and Herzegovina (Grant 27‐02‐35‐31288‐1/24).

## Conflicts of Interest

The authors declare no conflicts of interest.

## Data Availability

The data that support the findings of this study are available from the corresponding author upon reasonable request.
